# Achieving High Performance Electrode for Energy Storage with Advanced Prussian Blue-Drived Nanocomposites—A Review

**DOI:** 10.3390/ma16041430

**Published:** 2023-02-08

**Authors:** Dingyu Cui, Ronghao Wang, Chengfei Qian, Hao Shen, Jingjie Xia, Kaiwen Sun, He Liu, Cong Guo, Jingfa Li, Feng Yu, Weizhai Bao

**Affiliations:** 1 Institute of Advanced Materials and Flexible Electronics (IAMFE), School of Chemistry and Materials Science, Nanjing University of Information Science and Technology, Nanjing 210044, China; 2 Department of Materials Physics, School of Chemistry and Materials Science, Nanjing University of Information Science and Technology, Nanjing 210044, China; 3 Australian Centre for Advanced Photovoltaics, School of Photovoltaic and Renewable Energy Engineering, University of New South Wales, Sydney 2052, Australia

**Keywords:** Prussian blue analogues, electrode materials, energy storage, nanocomposites

## Abstract

Recently, Prussian blue analogues (PBAs)-based anode materials (oxides, sulfides, selenides, phosphides, borides, and carbides) have been extensively investigated in the field of energy conversion and storage. This is due to PBAs’ unique properties, including high theoretical specific capacity, environmental friendly, and low cost. We thoroughly discussed the formation of PBAs in conjunction with other materials. The performance of composite materials improves the electrochemical performance of its energy storage materials. Furthermore, new insights are provided for the manufacture of low-cost, high-capacity, and long-life battery materials in order to solve the difficulties in different electrode materials, combined with advanced manufacturing technology and principles. Finally, PBAs and their composites’ future challenges and opportunities are discussed.

## 1. Introduction

Environmental degradation and energy scarcity drive up demand for renewable energy. Energy storage and conversion is critical for renewable energy systems [1]. Governments all over the globe are becoming more conscious of the need of efficient green energy (solar energy, wind energy, and so on) and have made different efforts in green energy technology in accordance with global environmental standards. However, owing to material constraints, the performance of energy storage devices is far from meeting the needs due to the low performance of electrode materials [2]. In recent years, the family of Prussian blue (PB) and its analogue (PBA), a type of open frame structure materials, are widely used [3].

PB was discovered by chance by the Berlin artist Diesbach in 1704 and is thought to be the first synthetic metal–organic framework (MOF) reported in scientific literature. However, until the development of modern X-ray diffraction technology and its widespread application in structural determination, it lacked a clear crystal structure. PB is a mixed-valent ferrocyanide coordination polymer with a face-centered cubic structure composed of alternating Fe^2+^ and Fe^3+^ ions. Fe^2+^ and Fe^3+^ ions are surrounded by carbon and nitrogen atoms (Fe^2+^-C and Fe^3+^-N, respectively), leaving a large ion channel along the <100>direction. This unique structural arrangement in PB allows Fe to be replaced by other transition metal ions with different oxidation states, such as Cu^2+^, Mn^2+^, Ni2+, Zn^2+^, Co^2+^, and Co^3+^, resulting in PBA [4].

The unique structure of PB/PBA makes it a research hotspot [5]. Working as electrode materials in energy storage field, the family of PBA showed many advantages: (1) the unique open frame structure provides open channels allowing rapid anions and cations migration [6]; (2) the active site of the double redox reaction provides electrode with high specific charge/discharge capacity; (3) the manufacture of PBA is sample, inexpensive, and the form and type may be regulated manually. The excellent structure and performance of PBA make it widely used in various battery systems, such as Na-ion battery, Li-ion battery, K-ion battery, Ca-ion battery, Mg-ion battery, Al-ion battery, Zn-ion battery, etc. [7].

PBA’s unique structure provides several advantages in energy storage applications, yet it still has significant flaws. The main disadvantage of PBAs is their low crystal density. However, recent work found that composite materials combining PBA with other materials (such as oxides, sulfides, selenides, phosphates, borides, and carbides) can effectively neutralize the defects of the two materials [8]. When composite materials are used in energy storage, their cycle and electrochemical properties can improve significantly. As a result, it is possible to develop high-capacity, long-life, and cycle-stable energy storage materials [9]. As a result, electrode materials made by combining PBA with other materials have received considerable attention.

**Figure 1:**
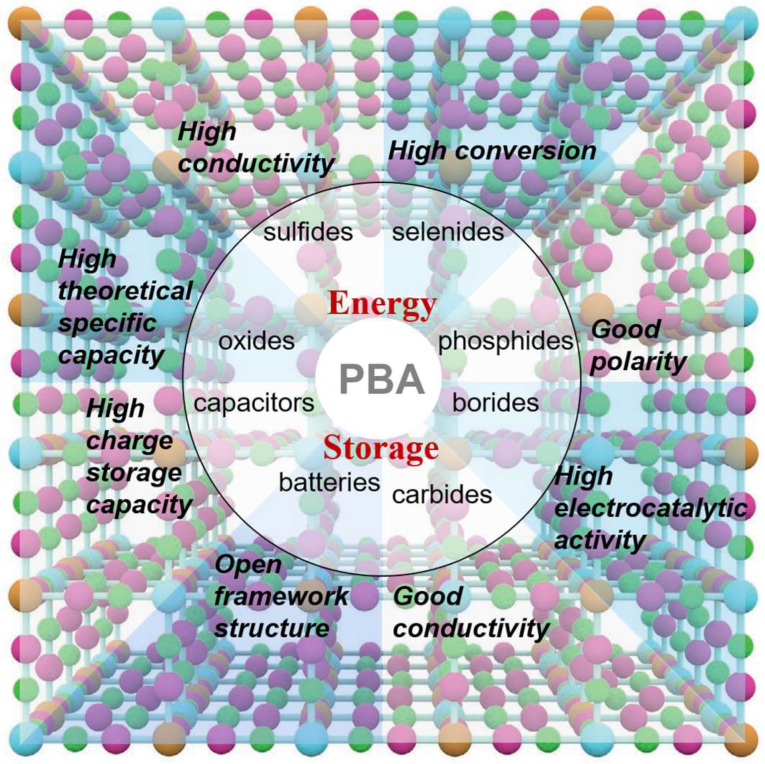
Schematic Diagram of PBA Application in Energy Storage Materials.

Unlike previous articles on the review of Prussian blue materials, we introduced the latest progress of PBAs-based electrode materials in this review, including oxides, sulfides, selenides, phosphates, borides, and carbides, under the guidance of advanced experimental methods and electrochemical characteristics (Table 1). We also discussed the formation of PBA in conjunction with other materials. The performance of composite materials improves the electrochemical performance of its energy storage materials [10]. Furthermore, new insights are provided for the manufacture of low-cost, high-capacity, and long-life battery materials in order to solve the difficulties in different electrode materials, combined with advanced manufacturing technology and principles [11]. Simultaneously, its development and use in supercapacitors and electrocatalysis are described (Figure 1). Finally, PBA and its composites’ future challenges and opportunities are discussed.

## 2. Prussian Blue and Its Analogues

### 2.1. Prussian Blue and Its Derivatives as Oxides

#### 2.1.1. Mechanism of Oxide Derived from Prussian Blue as Energy Storage Material

**Figure 2:**
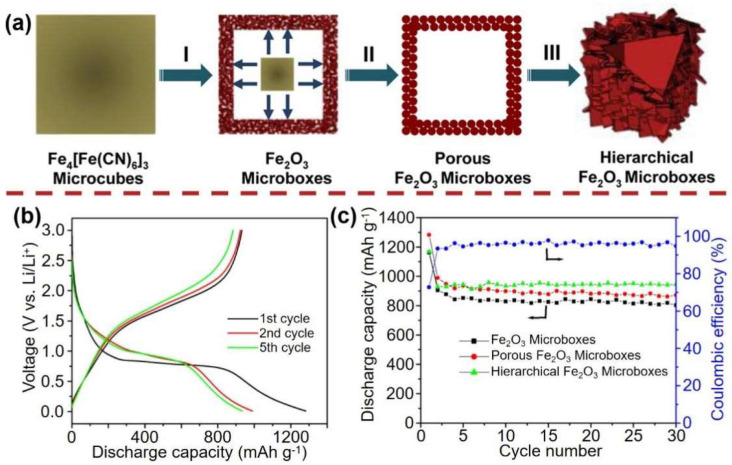
(**a**) Schematic illustration of the formation of hollow Fe_2_O_3_ microboxes and the evolution of the shell structure with the increasing calcination temperature. (**I** At temperatures below 350 °C, PB is converted to iron oxide in the near-surface region. **II** When the annealing temperature rises to 550 °C, the Fe_2_O_3_ microcapsules change into highly porous microcapsules. **III** When the annealing temperature rises to 650 °C, the highly porous shell is transformed into a layered shell composed of Fe_2_O_3_ nanoflakes.) (**b**) Discharge-charge voltage profiles of porous Fe_2_O_3_ microboxes obtained at 550 °C. (**c**) Cycling performance of Fe_2_O_3_ microboxes (350 °C), porous Fe_2_O_3_ microboxes (550 °C), and hierarchical Fe_2_O_3_ microboxes (650 °C), and Coulombic efficiency of porous Fe_2_O_3_ microboxes (550 °C) over the voltage range 0.01–3.0 V and Li/Li^+^ at the same current density of 200 mA g^−1^. Ref. [12] Copyright Journal of the American Chemical Society, ACS Publications.

Oxide derivatives with hollow nanostructures based on PB and PBA were investigated as anode materials for varous kind of rechargeable batteries system [12,13,30,31,32,33,34,35]. For example, PB cube can be derived as Fe_2_O_3_ [12]. These Fe_2_O_3_ microboxes have distinct physical architectures. The physical architectures of these Fe_2_O_3_ microboxes are unique. Their porous or hollow architectures may improve electrolyte diffusion and Li^+^ ions transport in the electrode, and this nano structure can effectively avoid volume change during the charging and discharging process [36]. At the same time, high crystallinity and hollow Fe_2_O_3_ microboxes provide structural stability during the long terms of charging and discharging process (Figure 2).

In addition to iron oxide, structure-related lithium storage properties can also be found in cobalt oxides derived from PBA. Co_3_O_4_ has been used in Li-ion battery because of its easy synthesis and high specific capacity. Because of the charge storage provided by the Faraday processes, cobalt oxide has attracted a lot of attention and has been studied for a long time as an energy storage material. Various forms of cobalt oxides are easy to synthesize and have very high theoretical capacitance. Co_3_O_4_ can be converted into CoOOH in an alkaline electrolyte, and then Co_3_O_4_ can be converted into CoO_2_ [37]. Although its theoretical capacitance is high, its practical capacitance is lower due to conductivity reduction and parasitic species formation during the long terms of charging and discharging process. Furthermore, it is noted that the expansion/contraction phenomenon limits the cycling life of cobalt oxide-based electrode. The main way to overcome these shortcomings is to design various high surface area morphologies.

**Figure 3:**
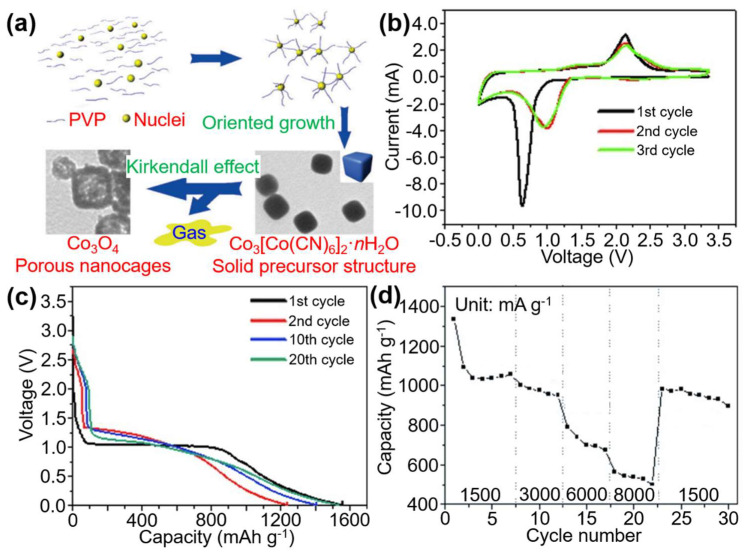
(**a**) Co_3_O_4_ porous nanocages have been prepared from Co_3_[Co(CN)_6_]_2_·nH_2_O truncated nano cubes based on the Kirkendall effect. (**b**) The first three CV curves of the Co_3_O_4_ porous nanocages based anode material recorded at a scan rate of 0.5 mV s^−1^. (**c**) The 1st, 2nd, 10th, and 20th discharge curves of the Co_3_O_4_ porous nanocage based anode materials at a current density of 300 mA g^−1^. (**d**) Rate capability of the as-prepared Co_3_O_4_ porous nanocages. Ref. [15] Copyright A European Journal, Wiley Online Library.

Hu et al. successfully demonstrated Co_3_O_4_ derived from Co_3_[Co(CN)_6_]_2_ PBA and obtained the porous Co_3_O_4_ nanocages [15]. Yi et al. found that porous Co_3_O_4_ nanocages had a better electrochemical performance as the anode of lithium solid cage. This nanostructure also has a high capacity for lithium storage [4]. As an energy storage material, it also shows many advantages: (1) the porous shell facilitates Li^+^ diffusion; (2) the bimodal pore size distribution and larger surface area reduce electrolyte resistance by shortening Li^+^ diffusion length, increasing electrolyte/electrode area of contact, and increasing electrolyte contact area; (3) coating amorphous carbon can adapt to cyclic-induced strain; (4) surface atoms and structural strain are enriched by small nanoparticles. These characteristics enable PBA-derived porous Co_3_O_4_ nanocages to provide significant benefits in the application of Lithium-ion batteries (LIBs), which may be exploited to create high-performance LIBs (Figure 3). All of these discoveries demonstrate that PBA oxide derivatives perform greatly as energy storage materials, and that they may be used to enhance the performance of LIBs in the future.

#### 2.1.2. Application of Oxide Derived from PBAs as Energy Storage Material

In order to pursue higher performance energy storage materials, researchers have carried out extensive exploration to further improve the new materials with a high capacity, stable cycle, and fast charging capacity. Because of their ease of availability, low cost, and high theoretical specific capacity, transition metal oxides are regarded as good energy storage materials [30]. The use of PBA-derived oxide nanomaterials will improve the optimization and design of the nanostructured electrode materials, improve ion diffusion ability and structural stability, and enable the production of high-performance energy storage materials. For instance, Zhang et al. [12] have explored the effect of Fe_2_O_3_ box secondary cells made of PB cubes at varying temperatures as anode materials for LIBs. Porous Fe_2_O_3_ micro particles composed of Fe_2_O_3_ nano particles can be easily synthesized by the oxidation and decomposition of PB nano cubes at high temperatures. When used as anode material of LIBs, the porous Fe_2_O_3_ nano cube obtained also shows the excellent cycle performance and high specific capacity (∼800 mA h g^−1^ at 200 mA g^−1^). In another experiment, researchers discovered that the electrochemical properties of Fe_2_O_3_ microboxes are also affected by the shell complexity [13]. The expandable synthesis of anisotropic hollow structures with numerous shell structures was demonstrated by the oxidative decomposition of PB microcube and crystal growth of iron oxide shell. The cycle stability of Fe_2_O_3_ microboxes with multiple shells is dramatically higher than that of Fe_2_O_3_ microboxes with single and double shells. When it is used as the anode material of LIBs, Fe_2_O_3_ has a well-defined hollow structure and microboxes with a layered shell, showing high specific capacity (∼950 mAh g^−1^ at 200 mA g^−1^) and outstanding cycle performance.

Meanwhile, the combination of metal oxides and carbon materials is a beneficial way to enhance the performance of LIBs. Using two PB/graphene foam precursors, Shao et al. [14] successfully prepared Fe_2_O_3_/graphene foam composites. The prepared Fe_2_O_3_/graphene foam material was used as a LIB’s independent electrode, combining the advantages of lead-derived metal oxides and graphene foam. It exhibited better lithium storage performance than pure Fe_2_O_3_ and graphene foam due to the synergistic effect of the two components. These research findings will help to develop and improve electrode materials for the storage of energy.

In addition, Hu et al. [15] suggested a new and simple method for creating porous Co_3_O_4_ nanocages based on the Kirkendall effect, which includes the thermal decomposition of PBA Co_3_[Co(CN)_6_]_2_ truncated nano cubes at 400 °C. These nanocages overcame the inherent disadvantage of Co_3_O_4_ anodes by exhibiting stability, high capacity, and excellent cycle efficiency. When applied to the electrode material of LIBs, the prepared Co_3_O_4_ porous nanocage showed excellent battery performance. At a current density of 300 mA g^−1^, it still had a capacity of 1465 mA h g^−1^ after 50 cycles. Metal oxides have a huge storage capacity; however, they suffer from significant volume fluctuations and have low conductivity when charged and discharged. Zhu et al. overcame this challenge by calcining preformed PBA Zn_3_[Co(CN)_6_]_2_ nanospheres to create self-assembled ZnO/Co_3_O_4_ nanocomposites [16]. These ZnO/Co_3_O_4_ nanocomposites have good cycling and lithium storing capacity. The rational design of the formed cluster structure, together with the synergistic impact of the bicomponent functional nanoparticle system, considerably improves the electrochemical performance of the electrode material generated from it. This special nanostructure has superior lithium storage capacity, which provides a new direction for further optimization of LIBs.

### 2.2. Prussian Blue and Its Derivatives as Sulfide

#### 2.2.1. Mechanism of Sulfide as Energy Storage Material

Metal sulfides have been shown to have higher conductivity, mechanical stability, thermal stability, and electrochemical activity than equivalent metal oxides in recent years [38,39,40]. Metal sulfides offer higher conductivity than comparable metal oxides, as well as a rich valence state, mechanical and thermal stability, all of which are beneficial to electrochemical performance [41]. Mixed metal sulfide is gaining popularity as a viable electrode material for electrochemical energy storage and conversion systems [42]. When compared to a single metal sulfide, mixed metal sulfide performs significantly better electrochemically [11,43], owing to better electronic conductivity and more diverse redox processes. Metal sulfide has emerged as a potential electrode material due to its extensive redox chemistry, high conductivity, and the synergistic action of two metal ions. Because of their better performance and widespread use in recent years, lithium sulfur batteries are a potential new energy storage technology that can fulfill the rising energy demand. A typical LIB is made up of a negative electrode, a positive electrode, an electrolyte, and a separator sandwiched between parallel electrodes [44,45,46]. The working principle of both devices is voltage-driven cation migration (Na^+^, Li^+,^ H^+^, K^+^, etc.) or anions (OH^−^, etc.) through electrolyte towards electrodes for reversible electrochemical reactions. At the same time, electrons flow via the external circuit to keep the charge balanced. These lithium batteries require appropriate and effective materials, particularly as matrix, sandwich, and laminated metal organic skeleton as a novel porous material. The chemical characteristics and physical architectures of electrode materials must be appropriate for an efficient energy storage system [47,48]. Metal–organic framework composites and MOF derivatives have a better performance, reducing the shortcomings of pure MOFs [49,50].

Previous research found that interstitial water promoted the insertion and extraction of multivalent ions. PBAs have excellent electrochemical performance because water weakens the electrostatic repulsion between ions, lowering the activation energy of ion diffusion and interface transfer [51]. Lithium ion is relatively easy to embed in PBAs for ion diffusion, whereas sodium or potassium are more difficult to embed due to defects that inhibit sodium/potassium diffusion and coordination water. The vacancy and water content in PBA will be significantly reduced through a well controlled crystallization process, so that the cycle capacity and capacity of sulfur ion battery (SIB) and LIB can be improved [52]. PBA and its sulfide derivatives play an important role in the application of energy storage materials because they provide sufficient gaps for the transfer of ions and electrolytes, as well as many active sites for the insertion of ions.

#### 2.2.2. Application of Sulfide as Energy Storage Material

PB and its sulfide derivatives are favored by many researchers because of their excellent performance, so PB and its sulfide derivatives have many applications in energy storage materials.

**Figure 4:**
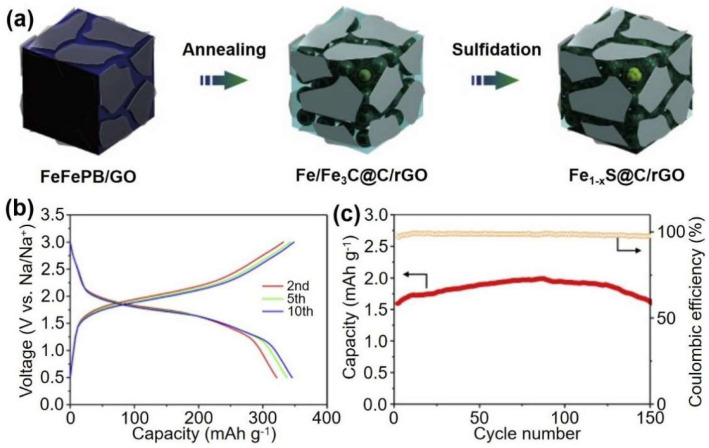
(**a**) Illustration of the formation process of Fe_1−x_S@C/rGO. (**b**) Galvanostatic discharge/charge curves of sodium ion full cells at different cycles at a current density of 200 mA g^−1^. (**c**) Cycle performance of sodium ion full cells at a current density of 200 mA g^−1^ (capacities are calculated based on anode mass). Ref. [17] Copyright Electrochimica Acta, Elsevier.

A graded iron sulfide nano cube was synthesized using PB as the starting material in a two-step in situ transformation process, and it was coated with several layers of graphene (Fe_1−x_S@C/rGO). When used as the anode of SIBs, the graded nano cube shows excellent rate capability of 323 mAh g^−1^ at a current density of 10 A g^−1^ [17]. For 150 cycles, the iron base sodium ion battery has a classification of Fe_1−x_S@C/rGO anode and the capacity of PB cathode is 323 mAh g^−1^. The Fe_1−x_S@C/rGO nano cube has good sodium ion storage performance due to the stable layered building structure and highly graphitized carbon obtained during the conversion process. The nano cube structure of graphene coating prevents the amalgamation of iron sulfide carbon core–shell nanoparticles and also adapts to the large volume expansion during the cycle. Carbon’s high crystallization degree conferred great electronic conductivity, enabled sodium ion accessibility, and enhanced mechanical durability on Fe_1−x_S@C/rGO nano cubes (Figure 4).

Zeng et al. created 3D hollow CoS_2_ nanoframes (HCSN) using partial anion replacement of Co-based PB nano cubes [53]. Because of its uniform broken hollow structure and excellent dispersion, the synthesized three-dimensional (3D) HCSN has a large specific surface area, leading to good electrochemical performance, such as the high specific capacitance of 568 F g^−1^ at 0.5 A g^−1^ and the excellent cycle stability of 88.3% specific capacitance retention after 5000 cycles. The HCSN supercapacitor has a great energy density of 32.3 Wh kg^−1^ and a power density of 4 kW kg^−1^. The HCSN also exhibit excellent structural stability and low density, indicating a potential application in energy storage materials.

The development of these new devices and their high performance show that PB sulfide derivatives play a significant role in the application of energy storage materials, as well as offering new ideas for the further development and utilization of sulfide in the future.

### 2.3. Prussian Blue and Its Derivatives as Selenide

#### 2.3.1. Mechanism of Selenide as Energy Storage Material

Recently, many researchers have become interested in transition metal selenides, which have the advantages of a high conversion rate and capacity [54]. Compared with metal sulfides and metal oxides, transition metal selenides have weak metal selenium ion bonds, which is conducive to the rapid transport of Li^+^/Na^+^ [55,56]. Metal selenides have the potential of cycle stability and excellent charge discharge specific capacity due to the powerful electronic conductivity of Se (1 × 10^−5^ S m^−1^) and weak electronegativity. Among the discovered cathode materials for sodium ion batteries, metal selenides with huge theoretical capacity are regarded as a promising candidate. However, metal selenide anodes are still plagued by poor electron conductivity, low initial coulomb efficiency, and rapid volume change during charging and discharging. In addition, the “shuttle effect of polyselenides” (shuttle of polyselenides dissolved between positive and negative electrodes) will lead to capacity degradation during the cycle. As a result, numerous mitigation measures have been implemented in order to alleviate these issues. MOF has recently been used as a precursor for preparing hollow structures of metal oxides and sulfides due to its good morphology, high surface area, and uniform porous structure. Because of its special size and shape dependence, PB and its analogues are composed of metal ions coordinated with rigid organic cyano groups and have been evolved for the design and preparation of various porous nanostructures [33,57].

#### 2.3.2. Application of Selenide as Energy Storage Material

PBA selenide derivatives can be used to build porous or hollow nanostructures with a high specific surface area and a unique shape that promotes electron transmission while avoiding significant volume expansion and thereby improving conductivity [58,59,60]. At the same time, by combining nanomaterials with different band gaps, it can promote the diffusion rate of Li^+^/Na^+^ and accelerate the surface reaction kinetics, which can form the design of bimetallic selenide architecture with unique interface effects. PBA selenide derivatives have a wide range of applications in energy storage materials. In addition, PBAs, as a MOF composed of metal centers and organic connectors, are available as self templates for the synthesis of porous materials [61]. MOF can limit and generate small size metal particles in situ, which can then be used to design metal selenides. Due to their excellent properties, many researchers are drawn to develop and use them.

**Figure 5:**
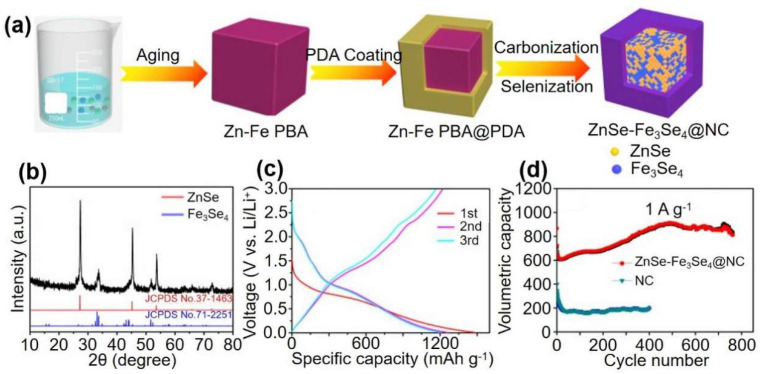
(**a**) Schematic diagram of synthesis process of the ZnSe-Fe_3_Se_4_@NC. (**b**) XRD pattern of ZnSe-Fe_3_Se_4_@NC. (**c**) Discharge and charge profiles of ZnSe-Fe_3_Se_4_@NC anode. (**d**) Long cycling performance of ZnSe-Fe_3_Se_4_@NC and NC anodes at 1 A g^−1^. Ref. [18] Copyright Journal of Alloys and Compounds, Elsevier.

**Figure 6:**
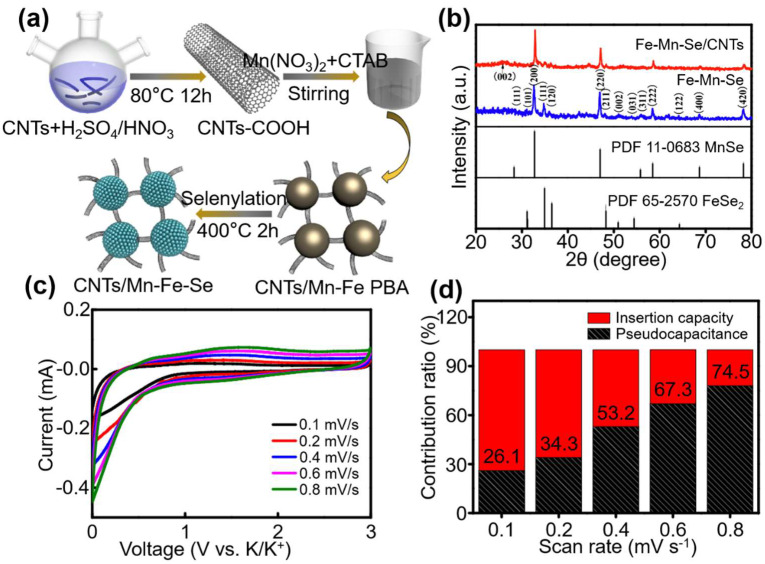
(**a**) Schematic representation of Mn-Fe-Se/CNTs composite. (**b**) XRD analysis of Mn-Fe-Se and Mn-Fe-Se/CNTs composites. (**c**) CV profiles at different scan rates. (**d**) Contribution ratio of capacitive current and diffusion-controlled current versus scan rate of Mn-Fe-Se/CNTs electrode for PIBs. Ref. [19] Copyright Chemical Engineering Journal, Elsevier.

Zhang et al. took full advantage of the porosity of PBA materials and the high capacity and high conversion of selenides, and used Zn-Fe PBA@polydopamine as a precursor to carbonization-selenization to design the microporous nanostructure ZnSe-Fe_3_Se_4_@NC successfully [18]. In addition, they investigated the electrochemical performance of N-doped Zn-Fe-Se heterostructures in LIBs. The ZnSeFe_3_Se_4_@NC anode exhibits excellent cycle stability and rate performance at 1 A g^−1^ after 723 cycles for LIBs (900 mA h g^−1^) because of the advantages of high conductivity carbon coating and porous structure (Figure 5). Previous research has shown that nanostructure fabrication and hybridization with carbonaceous materials are effective methods for mitigating mechanical strain caused by volume and structure changes during charge and discharge processes [62,63]. On the one hand, due to the short transmission path, reducing the electrochemical active materials to nano scale facilitates ions and electrons [64]. The carbonaceous reagent, on the other hand, can not only improve the transmittance of the electrode but also act as an effective buffer matrix to mitigate volume and structure changes during the cycle [65]. Wang et al. [19] successfully designed porous Mn-Fe-Se adhered/inserted with interlaced CNTs using a simple chemical precipitation approach and a one-step carbonization-selenization of the Mn-Fe PBA precursor process. Mn-Fe-Se/CNT exhibit remarkable electrochemical properties as well as high cycling and rate capabilities due to the synergistic effect of interlaced CNT (Figure 6). N-doped FeC@C box, which acts as the efficient polyselenide reservoir, is successfully created via a simple pyrolysis method using PB nano cubes as a precursor by Wang et al. [66]. Their results show that the PBA selenide derivative battery has high reversible specific capacity, better conduction rate, and cycle stability.

The above results indicate that PBA selenide is a promising electrode material for LIB. This result is expected to arouse people’s interest in energy storage and conversion of PBA selenide derivatives.

### 2.4. Prussian Blue and Its Derivatives as Phosphide

#### 2.4.1. Mechanism of Phosphide as Energy Storage Material

Transition metal phosphides are one of the candidates for LIBs polar materials, because they have good polarity, high catalytic activity, thermal stability, and chemical stability [67,68,69]. Therefore, it is critical to investigate the use of TMPs in Li-S batteries. Huang et al., using the density functional theory, showed that FeP can form a strengthening bond with polysulfides, promoting further redox conversion [70]. Cheng et al. [71] created double-layer Ni-Fe-P/N-doped carbon nanomaterials and demonstrated their stable cycle and excellent rate performance in LIBs. Hard carbon and soft carbon are the most common anode materials, but their capacities are limited (generally less than 300 mAh g^−1^) [72]. FeP has recently been considered as a potential candidate due to its good theoretical capacity (926 mAh g^−1^), medium operational potential, and environmental friendliness [73]. Nevertheless, major problems related to the non-negligible volume change of FeP particles and low inherent conductivity must be solved, which will greatly reduce the relationship between dynamics and cycle stability [74]. To address the previously mentioned tradeoff between specific capacity and cycle stability, particle size reduction can effectively decrease diffusion length while increasing sodium diffusion coefficient [75,76]. FeP nanoparticles (12 nm) anchored and uniformly dispersed on nitrogen-doped carbon frames (FeP@NC). It has a greater reversible capacity of 253.9 mAh g^−1^ under 2 A g^−1^ [77]. Considering the uniform distribution of Fe in the PB framework, it is expected that the phosphating/carbonization of PB will lead to the uniformly distributed of FeP nanoparticles in the carbon matrix.

#### 2.4.2. Application of Phosphide as Energy Storage Material

The excellent properties of PBA phosphide derivatives in energy storage materials make them favorable to many researchers, and many researchers have developed and applied them.

Song et al. [20] used Fe-Ni-P@nitrogen-doped carbon (Fe-Ni-P@NC) derived from Fe-Ni PBA, which is used as the efficient sulfur host of Li-S batteries. Fe-Ni-P particles not only effectively promote the conversion of LiPS, but also improve the adsorption of LiPS. Furthermore, the CN- of PBAs is easily converted into nitrogen-doped carbon during pyrolysis, which can improve the composite’s conductivity. Because of these benefits, LIBs/SIBs with S@Fe-Ni-P@NC composite cathodes demonstrated excellent electrochemical performance, excellent rate capability, and a stable cycle of 500 cycles at 1 ℃, and the capacity decay rate of each cycle is low, 0.08%.

**Figure 7:**
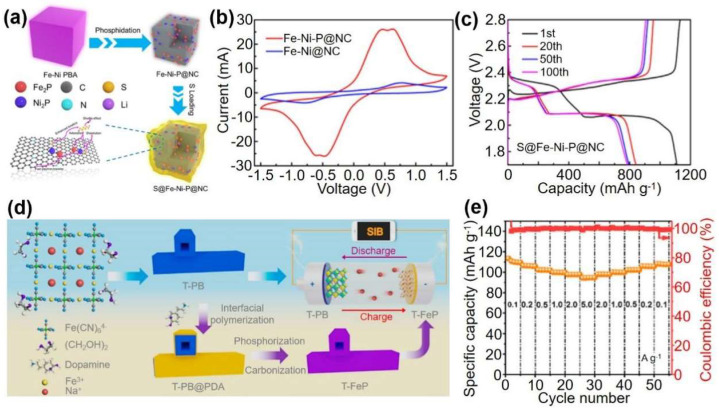
(**a**) Schematic diagram of the preparation of S@Fe-Ni-P@NC composites. (**b**) CV profiles of Fe-Ni-P@NC and Fe-Ni@NC in symmetric cell. (**c**) Galvanostatic charge–discharge curves.[20] (**d**) Schematic diagram for the synthesis of T-PB and T-FeP. (**e**) Rate capability at different current densities from 0.1 to 5.0 A g^−1^. Ref. [22] Copyright Journal of Power Sources, Elsevier.

By combining the tubular PB cathode with its derived phosphating anode, Jiang et al. demonstrated an excellent sodium ion battery [22]. Because of the tubular configuration, the PB has fast reaction kinetics and, thus, a high specific capacity of 94.4 mAh g^−1^ even at a high rate of 5.0 A g^−1^. PB-derived phosphides are characterized by encapsulating FeP nanoparticles in a conductive carbon matrix, thereby achieving excellent sodium energy storage. The as-assembled sodium-ion full cell has a record capacity of 105.3 mAh g^−1^ at 2.0 A g^−1^ and a long cycling lifetime (Figure 7). Their research proposes a homologous design strategy for outstanding sodium-ion full cells based on PB and its derivatives.

By making full use of the structural advantages of FeP, Shi et al. discovered a new binder-free anode material for high-performance sodium ion storage made of electrospun free-standing FeP@NPC film with FeP nanoparticles wrapped in 3D interconnected N, P-codoped carbon fiber [75]. The 3D-linked carbon fiber network acts as an electron/ion transport path, accelerating reaction kinetics while also adapting to the volume expansion of FeP during the sodium/disodium formation process. The unique structure design provides several structural advantages: the polyacrylonitrile nanofiber confines the growth of FeP nanoparticles during synthesis, reducing agglomeration of FeP; the structure has a high reversible capacity and cycle stability. This study lays the groundwork for a novel strategy for fabricating a phosphides-based high-performance anode for use in energy storage devices.

These studies and applications demonstrate that PBA phosphide derivatives perform exceptionally well in the application of energy storage materials. PBA phosphide derivatives will continue to be developed and applied in the future, with an eye toward their use in flexible electronic materials.

### 2.5. Prussian Blue and Its Derivatives as Boride

#### 2.5.1. Mechanism of Boride as Energy Storage Material

Metal borides’ electrochemical properties have been extensively researched due to their high chemical stability, low cost, conductivity, and unique electronic interaction between boron atoms and metal atoms [78]. Metal boride nanostructures can open up an entirely new dimension for harnessing these properties. Nanostructures can provide advantages such as increased surface area, easier access to exposed catalytic centers, faster reaction rate kinetics, tunable electronic structures, and lower charge transfer resistance [79,80]. Recent research has revealed that transition metal borides have the potential to be effective electrocatalysts for water splitting [81,82,83]. Bimetallic and ternary borides have been shown to have higher electrocatalytic activity than single-metal borides due to a synergistic effect [84,85]. Schuhmann et al. used operando X-ray absorption spectroscopy on ultrathin nickel boride nanosheets to demonstrate this aspect [82]. These studies clearly illustrate that the surface oxidation states change from Ni^2+^ to Ni^3+^ during the oxygen evolution reaction (OER) process, resulting in the formation of a NiO(OH) surface layer on a NiB@NiO(OH) core–shell structure. Amorphous cobalt boride nanosheets are also effective at OER electrocatalytic water splitting [86]. NiCoB and NiFeB nanosheet heterostructures with r-GO and single phase borated metal boride layers exhibit good performance in OER electrocatalysis [87,88]. The presence of heteroatoms is thought to reduce charge transfer resistance, increase the density of active catalytic sites, and ease electronic state regulation. As a type of MOF family, PBAs have been used as precursors or templates for metal hydroxides, phosphides, and sulfides. PBAs can be converted into various nanostructured metal borides by mild chemical reduction based on their different structures and metal ions. Many researchers worked on developing this material, which has excellent performance in the field of energy storage.

#### 2.5.2. Application of Boride as Energy Storage Material

PBA boride derivatives have been developed and utilized for their excellent properties in energy storage materials. He et al. synthesize amorphous Co-Ni-B-O nanosheets (CNBO-NSs) by chemically reducing bimetallic PBA (Co-Ni PBAs) with sodium borohydride [23]. The electrochemical catalytic activity and stability of the as-prepared CNBO-NSs are excellent. During 24 h operation, they can maintain a current density of 10 mA cm^−2^ at 140 mV over potential for hydrogen evolution and 300 mV for oxygen evolution. The formation of metal boron bonds, as well as an increase in surface area and conductivity in the CNBO-NS structure, are the factors that contribute to the increase in catalytic activity. This research can provide new insights for the application of MOF in the design of functional nanomaterials, as well as valuable insights for efficient electrolytic energy conversion and other aspects of crystallization engineering. As an efficient and durable electrocatalyst for OER, CoFe-based nanomaterials derived from PBA have increased specific surface area and rich catalytic active sites. Yang et al. [24] found that due to the adjustable chemical conformation and controllable 3D morphology, CoFe-B nano cube has a nano cube structure wrapped in ultra-thin nano sheets, and has excellent electrocatalytic performance and remarkable reaction kinetics in 1.0 m KOH aqueous solution. Furthermore, CoFe-B nano cubes have overpotentials of 261 and 338 mV at current densities of 10 and 200 mA cm^−2^, respectively, with a small Tafel slope of 61 mV dec1 for OER. The unique ultra-thin nano sheet wrapped nano cube structure is largely responsible for the excellent electrocatalysis performance. This work not only introduces a new method for using PBA, but it also introduces an efficient scheme for electrocatalysis.

### 2.6. Prussian Blue and Its Derivatives as Carbide

#### 2.6.1. Mechanism of Carbide as Energy Storage Material

Metal carbides are ideal electrode materials for electrochemical storage devices (such as LIB and the supercapacitor) because of their excellent stability, good conductivity, and high theoretical capacity [89,90]. Metal carbides have greater electronic conductivity and mechanical stability, as well as corrosion resistance, due to their special atomic structure in which carbon atoms are found in the voids between densely packed metallic host atoms [90,91]. Gogotis et al. proved that two-dimensional layered carbides can be used as anode materials for LIBs, and found that the Li^+^ storage mechanism is the intercalation and de intercalation of Li^+^ layers. Furthermore, Gogotsi and his colleagues demonstrated the potential of layered carbides as electrode materials for supercapacitors [92]. When PBAs are combined with carbides, two-dimensional layered carbides act as a binder and conductive additive to connect the nanoparticles, facilitating charge transfer and avoiding the significant drop in electrode conductivity that would otherwise occur. PBAs can increase interlayer space, electrolyte diffusion, and battery electrochemical activity, making them perfect for all aspects of the energy storage sector. In addition, metal carbides and carbon-encapsulated metal/metal alloys exhibit outstanding electrocatalytic activity due to the synergistic impact between carbon materials and metals. PB/PBA nanoparticles with uniform element distribution and abundant cyanide ligands are also being investigated as chemical precursors for the preparation of catalytic metal carbides and carbon-encapsulated metal/metal alloys [93]. Because of the superior features of metal carbides, they offer significant benefits in the fields of energy storage and electrocatalysis, and they are frequently investigated and implemented in practice.

#### 2.6.2. Application of Carbide as Energy Storage Material

**Figure 8:**
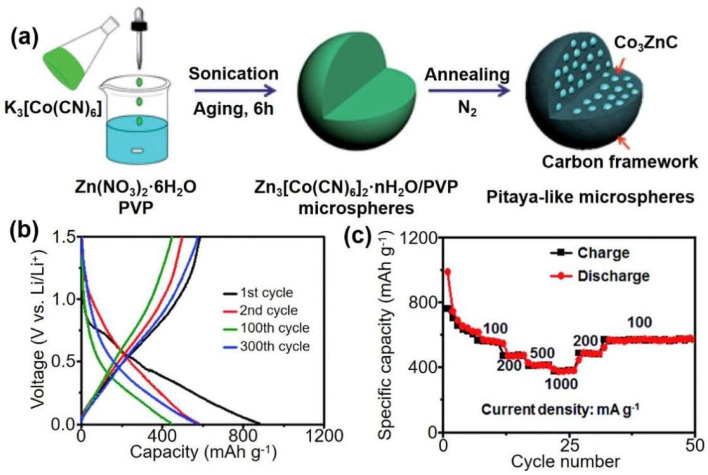
(**a**) Schematic illustration of the formation process of pitaya-like microspheres. (**b**) Charge/discharge profiles of pitaya-like microspheres as anode material of LIBs at 100 mA g^−1^. (**c**) Rate performance of pitaya-like microspheres at various current densities range from 100 to 1000 mA g^−1^. Ref. [25] Copyright Journal of Materials Chemistry A, pubs.rsc.org.

It is an effective method to use PBA as the precursor and provide two metal elements at the same time to form bimetallic carbide and carbon skeleton nanocomposites with distinct structures. Ma et al. developed a method of implanting uniformly distributed carbide nanoparticles into a spherical porous carbon framework to form microspheres similar to pitaya [25]. The synthesized pitaya-shaped microspheres can effectively buffer volume changes and prevent Co_3_ZnC nanoparticles from aggregating during the charging/discharging process of LIBs due to their unique composition and structural characteristics. The porous carbon framework allows unimpeded electron transmission and Li diffusion, and restricts the thin solid electrolyte interface layer to the outer surface of the carbon shell. After 300 charge/discharge cycles, the anodes in LIBs deliver a high capacity of 608 mA h g^−1^ at 100 mA g^−1^ and ultrahigh cyclic stability and rate performance with a capacity of 423 mA h g^−1^ even after 1150 consecutive cycles at 1000 mA g^−1^ (Figure 8).

The thermal decomposition of PB produced Fe/Fe_3_C without altering its original morphology, but the surface area and porosity increased significantly [94]. Due to the presence of metallic iron, each of these Fe/Fe_3_C nanoparticles is uniformly coated with several layers of graphite carbon, which enhances its stability and electronic conductivity. Kumar et al. reported for the first time a one-step method for manufacturing the unique superstructure of carbon-encapsulated Fe/Fe_3_C nanocomposites for supercapacitor electrodes [26]. Nanocomposites with carbon encapsulation have excellent cycle stability and no capacitance decay after 20000 CV cycles. Asymmetric supercapacitors exhibit good electrochemical performance in terms of capacitance, energy, power density, and cycle stability when assembled, which is practical for future applications. The detailed investigation of PBA carbide derivatives provides us with a new idea in the research and development of energy storage materials.

## 3. Application

### 3.1. Application of PBAs in Batteries

PB/PBA and its derivatives are unique materials. They have recently regained much interest due to their unique characteristics, and some of them are ideal for energy storage. (1) They have theoretically high specific capacity [95]. (2) Their geometric form varies extremely little throughout the ion insertion process, leading in a cycle life that is astonishingly lengthy [96]. (3) Coprecipitation processes utilizing water precursors create these chemicals, resulting in easy and low-cost preparation procedures [97]. (4) Their cubic geometry, wide nanopore channels, and open framework structure enable ions to conduct rapidly and efficiently [98].

PBAs are suitable as ion hosts due to their ease of preparation, tunable composition/structure, and unique open framework, particularly for large cations such as Li^+^ and K^+^ [99]. In order to put PBA into large-scale, low-cost, high-performance practical applications, it is necessary to further improve the rate capability and cycle life, and significantly improve the ion conductivity and electronic conductivity of PBAs [100]. Not only does the increased size of multivalent cations have an adverse effect on cation diffusion, but so does the increased electrostatic repulsion. In this case, increasing the amount of vacancy and co-ordination water can reduce the electrostatic force and improve the transport and storage of cations. A macroporous morphology, on the other hand, can facilitate electrolyte access and accelerate cation diffusion. Combining or coating conductive carbons such as graphene, carbon nanotubes, and carbon black can significantly improve PBA’s limited electric conductivity, which hinders its electrochemical performance. Furthermore, those interested in designing multi-component metal oxides as catalysts for metal-air batteries can take advantage of PBAs’ simple preparation process and composition diversity. Considering all these, it is believed that the basic research on PBA materials will be helpful to develop high-performance battery electrode materials.

PBAs are useful templates for producing different metal oxides, metal sulfides, metal selenides, metal phosphides, metal borides, and metal carbides in nanostructure synthesis. The electrode materials derived from Prussian blue nanocomposites may fully use the benefits of all parties to create a new generation of high-performance ordering devices. The use of PBAs in multivalent batteries is a novel research topic that is still in its early stages. Future research will be required, such as the PBA storage mechanism and the construction of a novel PBA framework with a lengthy life cycle. PB/PBAs and their derivatives are being investigated as novel electrode materials. These researches provide new ideas and directions for the development of new battery materials in the future.

### 3.2. Application of PBAs in Capacitors

PB/PBAs and their derivatives have been considered as potential candidates for efficient supercapacitors. The intrinsic metal center in the original PB/PBA and its derivatives provides redox active sites for the Faraday reaction, increasing charge storage capacity. For the first time, Yi et al. successfully developed a new method of CoPBA@Ni(OH)_2_ core–shell structure. This method uses in situ etching and growth to cover the surface of a CoPBA nano cube with a Ni(OH)_2_ nano sheet before vulcanizing it into a CoS_2_@NiS_2_ hierarchical porous structure. Because of its excellent structure, this CoS_2_@NiS_2_ composite material has become a high-performance supercapacitor. PBA, as the central core of the cubic skeleton in this structure, can provide an effective electron conduction path to reduce resistance. In addition, it effectively prevents Ni(OH)_2_ and CoS_2_@NiS_2_ agglomeration, and its large surface area and mesoporous structure reduce ion diffusion paths, improving cycle stability. This material has a very high specific capacitance value of 1731.2 F g^−1^ at 1 A g^−1^ and cycle performance of 87.1% retention after 5000 cycles [27].

Although PB/PBA showed promising results, the poor conductivity of the original PB/PBA frame largely destroyed the capacitance. Many researchers have explored the use of conductive carbon substrates (such as graphene, activated carbon, or carbon fiber) to give PB/PBA frames higher conductivity, thereby improving conductivity and stability. For example, Wang et al. successfully fabricated high-quality graphene@PB (G@PB) nanocomposite sheets using a one-step in situ hydrothermal method in which uniform PB nanoparticles completely covered both sides of graphene sheets by controlling the etching of the raw material and the growth of the target products [28]. Graphene not only prevents the aggregation of PB nanoparticles, but also provides a conductive network for rapid electron transport. The G@PB-5 hybrid composite, in particular, shows the highest capacitance of 388.09 F g^−1^ at a current density of 1 A g^−1^, as well as enhanced rate capability and long-term stability with 97.2% retention over 5000 cycles and coulombic efficiency of nearly 100%. By combining the optimized nanocomposite electrode with the active carbon negative electrode, an asymmetric supercapacitor with a reversible working voltage of 2.0 V was constructed. The G@PB-5 nanocomposite sheets are promising for energy-storage hybrid electrodes due to their high electrochemical performances.

In addition to using the original PB/PBA as the supercapacitor electrode, researchers also studied the capacitive charge storage capacity of its derived nanostructures and the resulting energy storage density of asymmetric and hybrid supercapacitor devices. Yu and co-workers proposed a new structure-induced anisotropic chemical etching/anion exchange method for converting Ni-Co PBA nano cubes into tunable NiS nano frameworks [29]. The reaction of Ni Co PBA nano cube template with Na_2_S in solution leads to the formation of a well-defined NiS nano framework. The different reactivity of the edge and plane surfaces of the Ni Co PBA nano cube is discovered to be the key factor in the formation of the NiS nano frame. Due to the structural advantages of 3D open structure, namely a high specific surface area, the small size of primary nanoparticles, and good structural robustness, the derived NiS nano framework shows excellent electrochemical performance for supercapacitors.

Due to its superior properties, PB/PBA is widely employed in the field of energy storage devices. Although PB/PBA have good qualities, their conductivity is rather low, which poses a significant barrier to their implementation. PB/PBA derivatives have a larger surface area, conductivity, and more open diffusion channels than the original PB/PBA with porous and hollow morphology. These have a substantial impact on electrochemical performance. As a result, PB/PBA has been frequently employed as a precursor or sacrificial template in the production of derivatives. Adding conductive fillers, such as carbon materials and other metal compounds, improves the conductivity of PB and PBA materials. When compared to its original form, the addition of functional elements can improve its electrochemical performance, allowing high-performance supercapacitors to be created. We look forward to further research and development of PB/PBA in future supercapacitors.

## 4. Outlook

Because of their unique chemical and physical properties, PBAs are widely used as electrode materials for electrochemical energy storage and conversion. We found that when PBA is combined with various compounds to use electrodes as energy storage materials, its unique open frame structure allows ions to pass through quickly, solving the low conductivity problem of various compounds and greatly improving the conductivity of the energy storage system. On the other hand, PBA can endow materials with high capacity and alleviate the problems of effective material loss, volume expansion or capacity attenuation caused by material dissolution in electrolyte.

For the synthesis of PBAs, the key is to form a hollow structure, so that the formed PB/PBA template or the precursor and the final product can be evenly distributed with thermodynamic stability. Second, researchers still do not know much about the nucleation and crystallization processes of PBAs materials due to differences in nucleation rates and solubility precipitation constants. As a result, precisely controlling the microstructure, particularly the ion content, coordination water content, and lattice defects, is difficult. Further in situ studies should be carried out to fully understand its structure, grasp the synthesis mechanism, and accurately control the synthesis results in order to better understand its growth kinetics and control its synthesis.

At the same time, the hollow structure of PBA is an excellent catalytic structure because it can provide a larger catalytic interface area, which improves electrocatalytic performance significantly. To reduce the activation energy and improve the conversion rate in the important hydrogen evolution reaction, oxygen evolution reaction, and oxygen evolution reduction reaction, an electro catalyst with high efficiency, durability, low cost, and sustainability is usually required. Metal phosphides, sulfides, and borides derived from PBA can be used for electrocatalytic hydrogen evolution in acidic and alkaline solutions, according to the findings. There is currently little research on the use of PBA as a catalyst, and researchers can further investigate its use as a catalyst.

In the future, introducing new elements into PBA-derivative materials and studying their modified functions will be the brand-new research direction of PBA-based electrode materials in the field of energy storage. In terms of increased electrochemical surface area, the morphology development of PBA-derived materials holds great promise. PBA-derived materials can also be formed into a variety of two-dimensional and three-dimensional hollow, porous, and core–shell forms, making full use of their open framework to build channels conducive to ion transport, which is also a future way to improve the electrochemical performance of PBA materials in various ion battery electrodes.

We believe that the unique structure of PBA makes it widely used in the future electronic device applications. To fully investigate the properties of PBA, it will be a future research hotspot to develop high-performance materials or make composites with PBA as the precursor.

## 5. Conclusions

We introduced in depth the structure and properties of PB/PBA, as well as spectacular examples of their interaction with other types of chemicals, in this work. Based on this assumption, we investigated anode materials (oxides, sulfides, selenides, phosphides, borides, and carbides) based on Prussian blue analogues (PBAs), which have gained widespread recognition in the field of energy conversion and storage due to their distinct properties (including high theoretical specific capacity, environmental protection, and low cost). We discovered that PBA’s distinctive open frame structure may give open channels for ions, which is favorable to fast ion conduction, and the active sites of the redox reaction provide high-capacity materials via the research of numerous PBA electrode materials. PBA is a widely used electrode material with considerable promise for future improvement. Composite materials synthesized with PBA as a precursor fully integrate the great properties of diverse materials, which is the direction of constructing high-performance energy storage materials. These PBA composites outperform in every way. Their distinctive open frame construction and superior performance provide significant benefits in the production of high-performance batteries, novel supercapacitors, and electrocatalysts. These novel composite materials, which use PBA as a precursor, offer a wide range of possibilities in future flexible electronic devices.

## Figures and Tables

**Table 1:** The electrochemical performance of PB and its derivatives for energy storage.

Precursors	Structures	Specific Capacity	Applications	Ref.
**PB**	Fe_2_O_3_	~800 mA h g^−1^ at 200 mA g^−1^	LIBs	[12]
**PB**	Multi shell Fe_2_O_3_	~950 mA h g^−1^ at 200 mA g^−1^	LIBs	[13]
**PB/graphene**	Fe_2_O_3_/graphene	~654 mA h g^−1^ at 100 mA g^−1^	LIBs	[14]
**Co_3_[Co(CN)_6_]_2_ PBA**	Co_3_O_4_ nanocages	~1465 mA h g^−1^ at 300 mA g^−1^	LIBs	[15]
**Zn_3_[Co(CN)_6_]_2_ PBA**	ZnO/Co_3_O_4_ nanocomposite cluster	~957 mA h g^−1^ at 100 mA g^−1^	LIBs	[16]
**PB**	Fe_1−x_S@C/rGO	~323 mA h g^−1^ at 10 A g^−1^	SIBs	[17]
**PB**	Hollow CoS_2_ nanoframes	568 F g^−1^ at 0.5 A g^−1^	capacitor	[17]
**Zn-Fe PBA@polydopamine**	ZnSe-Fe_3_Se_4_@NC	~900 mA h g^−1^ at 1 A g^−1^	LIBs	[18]
**Mn-Fe PBA**	Mn-Fe-Se/CNT	~411 mA h g^−1^ at 800 mA g^−1^	PIBs/SIBs	[19]
**Fe-Ni PBA**	S@Fe-Ni-P@NC	~470.8 mA h g^−1^ at 1 C	LIBs/SIBs	[20]
**Fe_4_[Fe(CN)_6_]_3_**	Fe/Fe_3_C@N-rGO		Electroctalysts	[21]
**PB**	FeP nanoparticles	~105.3 mAh g^−1^ at 2.0 A g^−1^	LIBs	[22]
**Co-Ni PBA**	CNBO-NSs		Electroctalysts	[23]
**PBA**	CoFe-B nano cubes		Electroctalysts	[24]
**PBA**	pitaya-like microspheres	~608 mA h g^−1^ at 100 mA g^−1^	LIBs	[25]
**PB**	Fe/Fe_3_C nanocomposites	223 F g^−1^ at 10 mV s^−1^	capacitor	[26]
**CoPBA**	CoPBA@Ni(OH)_2_	87.1%/5000 cycles	capacitor	[27]
**PB**	G@PB-5	97.2%/5000 cycles	capacitor	[28]
**Ni-Co PBA**	NiS nanoframes		capacitor	[29]

## References

[1] Dunn B., Kamath H., Tarascon J.-M. (2011). Electrical Energy Storage for the Grid: A Battery of Choices. Science.

[2] Mao Y., Chen Y., Qin J., Shi C., Liu E., Zhao N. (2019). Capacitance controlled, hierarchical porous 3D ultra-thin carbon networks reinforced prussian blue for high performance Na-ion battery cathode. Nano Energy.

[3] Ji B., Yao W., Zheng Y., Kidkhunthod P., Zhou X., Tunmee S., Sattayaporn S., Cheng H.-M., He H., Tang Y. (2020). A fluoroxalate cathode material for potassium-ion batteries with ultra-long cyclability. Nat. Commun..

[4] Song X., Song S., Wang D., Zhang H. (2021). Prussian Blue Analogs and Their Derived Nanomaterials for Electrochemical Energy Storage and Electrocatalysis. Small Methods.

[5] Feng Y., Wang X., Dong P., Li J., Feng L., Huang J., Cao L., Feng L., Kajiyoshi K., Wang C. (2019). Boosting the activity of Prussian-blue analogue as efficient electrocatalyst for water and urea oxidation. Sci. Rep..

[6] Simonov A., De Baerdemaeker T., Boström H.L., Gomez M.R., Gray H., Chernyshov D., Bosak A., Bürgi H.-B., Goodwin A. (2020). Hidden diversity of vacancy networks in Prussian blue analogues. Nature.

[7] Armand M., Tarascon J.M. (2008). Building better batteries. Nature.

[8] Bao W., Wang R., Qian C., Li M., Sun K., Yu F., Liu H., Guo C., Li J. (2022). Photoassisted High-Performance Lithium Anode Enabled by Oriented Crystal Planes. ACS Nano.

[9] Qian C., Wang R., Sun K., Bao W. (2022). Dimensional engineering of anode materials for high performance potassium ion hybrid capacitor—A review. Int. J. Energy Res..

[10] Wu X., Hong J., Shin W., Ma L., Liu T., Bi X., Yuan Y., Qi Y., Surta T., Huang W. (2019). Diffusion-free Grotthuss topochemistry for high-rate and long-life proton batteries. Nat. Energy.

[11] Shen L., Yu L., Wu H., Yu X.-Y., Zhang X., Lou X.W.D. (2015). Formation of nickel cobalt sulfide ball-in-ball hollow spheres with enhanced electrochemical pseudocapacitive properties. Nat. Commun..

[12] Zhang L., Wu H., Madhavi S., Hng H., Lou X. (2012). Formation of Fe_2_O_3_ Microboxes with Hierarchical Shell Structures from Metal–Organic Frameworks and Their Lithium Storage Properties. J. Am. Chem. Soc..

[13] Zhang L., Wu H., Xu R., Lou X. (2013). Porous Fe_2_O_3_ nanocubes derived from MOFs for highly reversible lithium storage. CrystEngComm.

[14] Shao J., Feng J., Zhu M., Zhou H., Yuan A. (2019). Prussian blue derived metal oxides/graphene foam as anode materials for high-performance lithium-ion batteries. J. Mater. Sci. Mater. Electron..

[15] Hu L., Yan N., Chen Q., Zhang P., Zhong H., Zheng X., Li Y., Hu X. (2012). Fabrication Based on the Kirkendall Effect of Co_3_O_4_ Porous Nanocages with Extraordinarily High Capacity for Lithium Storage. Chem. A Eur. J..

[16] Zhu D., Zheng F., Xu S., Zhang Y., Chen Q. (2015). MOF-derived self-assembled ZnO/Co_3_O_4_ nanocomposite clusters as high-performance anodes for lithium-ion batteries. Dalton Trans..

[17] Xiang J., Liu Z., Song T. (2018). Hierarchical iron sulfide-graphene nanocubes consisting of multiple nanoparticles with superior sodium ion storage properties. Electrochim. Acta.

[18] Zhang S., Wang Z., Hu X., Zhu R., Liu X., Wang H. (2021). Building Zn-Fe bimetal selenides heterostructures caged in nitrogen-doped carbon cubic for lithium and sodium ion batteries. J. Alloys Compd..

[19] Wang J., Wang B., Liu X., Bai J., Wang H., Wang G. (2020). Prussian blue analogs (PBA) derived porous bimetal (Mn, Fe) selenide with carbon nanotubes as anode materials for sodium and potassium ion batteries. Chem. Eng. J..

[20] Song C., Jin Q., Zhang W., Hu C., Bakenov Z., Zhao Y. (2021). Prussian blue analogs derived Fe-Ni-P@nitrogen-doped carbon composites as sulfur host for high-performance lithium-sulfur batteries. J. Colloid Interface Sci..

[21] Liu Y., Wang H., Lin D., Zhao J., Liu C., Xie J., Cui Y. (2017). A Prussian blue route to nitrogen-doped graphene aerogels as efficient electrocatalysts for oxygen reduction with enhanced active site accessibility. Nano Res..

[22] Jiang M., Ren L., Hou Z., Hua W., Lei D., Cao Y., Zhang Y., Wang J.-G. (2023). A superior sodium-ion battery based on tubular Prussian blue cathode and its derived phosphide anode. J. Power Sources.

[23] He T., Nsanzimana J., Qi R., Zhang J.-Y., Miao M., Yan Y., Qi K., Liu H., Xia B. (2018). Synthesis of amorphous boride nanosheets by the chemical reduction of Prussian blue analogs for efficient water electrolysis. J. Mater. Chem. A.

[24] Yang Y.X., Li B., Zhang Q., Guo W., Wang X., Li L., Luo H., Li N. (2021). Prussian Blue Analogues–Derived CoFe–B Nanocubes with Increased Specific Surface Area and Modulated Electronic Structure as Enhanced Oxygen Evolution Electrocatalysts. Energy Technol..

[25] Ma L., Chen T., Zhu G., Hu Y., Lu H., Chen R., Liang J., Tie Z., Jin Z., Liu J. (2016). Pitaya-like microspheres derived from Prussian blue analogues as ultralong-life anodes for lithium storage. J. Mater. Chem. A.

[26] Kumar A., Das D., Sarkar D., Patil S., Shukla A. (2020). Supercapacitors with prussian blue derived carbon encapsulated Fe/Fe_3_C nanocomposites. J. Electrochem. Soc..

[27] Wang S.-C., Xiong D., Chen C., Gu M., Yi F.-Y. (2020). The controlled fabrication of hierarchical CoS2@NiS2 core-shell nanocubes by utilizing prussian blue analogue for enhanced capacitive energy storage performance. J. Power Sources.

[28] Wang S.-C., Gu M., Pan L., Xu J., Han L., Yi F.-Y. (2018). The interlocked in situ fabrication of graphene@prussian blue nanocomposite as high-performance supercapacitor. Dalton Trans..

[29] Yu X.Y., Yu L., Wu H., Lou X. (2015). Formation of nickel sulfide nanoframes from metal–organic frameworks with enhanced pseudocapacitive and electrocatalytic properties. Angew. Chem..

[30] Wu F., Bai J., Feng J., Xiong S. (2015). Porous mixed metal oxides: Design, formation mechanism, and application in lithium-ion batteries. Nanoscale.

[31] Feng Y., Yu X.-Y., Paik U. (2016). Formation of Co_3_O_4_ microframes from MOFs with enhanced electrochemical performance for lithium storage and water oxidation. Chem. Commun..

[32] Yan N., Hu L., Li Y., Wang Y., Zhong H., Hu X., Kong X., Chen Q. (2012). Co_3_O_4_ Nanocages for High-Performance Anode Material in Lithium-Ion Batteries. J. Phys. Chem. C.

[33] Yu H., Fan H., Yadian B., Tan H., Liu W., Hng H., Huang Y., Yan Q. (2015). General Approach for MOF-Derived Porous Spinel AFe_2_O_4_ Hollow Structures and Their Superior Lithium Storage Properties. ACS Appl. Mater. Interfaces.

[34] Liu L., Hu Z., Sun L., Gao G., Liu X. (2015). Controlled synthesis and enhanced electrochemical performance of Prussian blue analogue-derived hollow FeCo_2_O_4_ nanospheres as lithium-ion battery anodes. RSC Adv..

[35] Huang G., Zhang L., Zhang F., Wang L. (2014). Metal–organic framework derived Fe_2_O_3_@NiCo2O4 porous nanocages as anode materials for Li-ion batteries. Nanoscale.

[36] Wang R., Sun K., Liu H., Qian C., Li M., Zhang Y., Bao W. (2022). Integrating a redox-coupled FeSe_2_/N–C photoelectrode into potassium ion hybrid capacitors for photoassisted charging. J. Mater. Chem. A.

[37] Nguyen T., Montemor M.D.F. (2019). Metal Oxide and Hydroxide–Based Aqueous Supercapacitors: From Charge Storage Mechanisms and Functional Electrode Engineering to Need-Tailored Devices. Adv. Sci..

[38] Rui X., Tan H., Yan Q. (2014). Nanostructured metal sulfides for energy storage. Nanoscale.

[39] Gao M.-R., Xu Y.-F., Jiang J., Yu S.-H. (2013). Nanostructured metal chalcogenides: Synthesis, modification, and applications in energy conversion and storage devices. Chem. Soc. Rev..

[40] Zheng X.-L., Song J.-P., Ling T., Hu Z., Yin P.-F., Davey K., Du X.-W.K., Qiao S.-Z. (2016). Strongly Coupled Nafion Molecules and Ordered Porous CdS Networks for Enhanced Visible-Light Photoelectrochemical Hydrogen Evolution. Adv. Mater..

[41] Cai P., Huang J., Chen J., Wen Z. (2017). Oxygen-containing amorphous cobalt sulfide porous nanocubes as high-activity electrocatalysts for the oxygen evolution reaction in an alkaline/neutral medium. Angew. Chem..

[42] Wang R., Zhang Y., Sun K., Qian C., Bao W. (2022). Emerging green technologies for recovery and reuse of spent lithium-ion batteries—A review. J. Mater. Chem. A.

[43] Chen H., Jiang J., Zhang L., Wan H., Qi T., Xia D. (2013). Highly conductive NiCo_2_S_4_ urchin-like nanostructures for high-rate pseudocapacitors. Nanoscale.

[44] Zhang Q., Uchaker E., Candelaria S., Cao G. (2013). Nanomaterials for energy conversion and storage. Chem. Soc. Rev..

[45] Rui X., Yan Q., Skyllas-Kazacos M., Lim T. (2014). Li_3_V_2_(PO4)_3_ cathode materials for lithium-ion batteries: A review. J. Power Sources.

[46] Zhao X., Sánchez B., Dobson P., Grant P. (2011). The role of nanomaterials in redox-based supercapacitors for next generation energy storage devices. Nanoscale.

[47] Yuan B., Sun F., Li C., Huang W., Lin Y. (2019). Formation of Prussian blue analog on Ni foam via in-situ electrodeposition method and conversion into Ni-Fe-mixed phosphates as efficient oxygen evolution electrode. Electrochim. Acta.

[48] Huang S., Wang H., Zhang Y., Wang S., Chen Z., Hu Z., Qian X. (2018). Prussian blue-derived synthesis of uniform nanoflakes-assembled NiS_2_ hierarchical microspheres as highly efficient electrocatalysts in dye-sensitized solar cells. RSC Adv..

[49] Du M., Li Q., Zhao Y., Liu C.-S., Pang H. (2020). A review of electrochemical energy storage behaviors based on pristine metal–organic frameworks and their composites. Coord. Chem. Rev..

[50] Cao X., Tan C., Sindoro M., Zhang H. (2017). Hybrid micro-/nano-structures derived from metal–organic frameworks: Preparation and applications in energy storage and conversion. Chem. Soc. Rev..

[51] Tang Y., Hu J., Tao H., Li Y., Li W., Li H., Zhou M., Wang K., Jiang K. (2022). Rational design of Prussian blue analogues as conversion anodes for lithium-ion batteries with high capacity and long cycle life. J. Alloys Compd..

[52] Bao W., Wang R., Qian C., Zhang Z., Wu R., Zhang Y., Liu F., Li J., Wang G. (2021). Porous Heteroatom-Doped Ti_3_C_2_Tx MXene Microspheres Enable Strong Adsorption of Sodium Polysulfides for Long-Life Room-Temperature Sodium–Sulfur Batteries. ACS Nano.

[53] Zeng X., Yang B., Li X., Yu R. (2017). Three-dimensional hollow CoS_2_ nanoframes fabricated by anion replacement and their enhanced pseudocapacitive performances. Electrochim. Acta.

[54] Zhou D., Liu Y., Song W.-L., Li X., Fan L.-Z., Deng Y. (2017). Three-dimensional porous carbon-coated graphene composite as high-stable and long-life anode for sodium-ion batteries. Chem. Eng. J..

[55] Yang T., Liu Y., Yang D., Deng B., Huang Z., Ling C., Liu H., Wang G., Guo Z., Zheng R. (2019). Bimetallic metal-organic frameworks derived Ni-Co-Se@C hierarchical bundle-like nanostructures with high-rate pseudocapacitive lithium ion storage. Energy Storage Mater..

[56] Li J., Yan D., Lu T., Yao Y., Pan L. (2017). An advanced CoSe embedded within porous carbon polyhedra hybrid for high performance lithium-ion and sodium-ion batteries. Chem. Eng. J..

[57] Zhang L., Wu H., Lou X. (2013). Metal–organic-frameworks-derived general formation of hollow structures with high complexity. J. Am. Chem. Soc..

[58] Ali Z., Asif M., Huang X., Tang T., Hou Y. (2018). Hierarchically porous Fe_2_CoSe_4_ binary-metal selenide for extraordinary rate performance and durable anode of sodium-ion batteries. Adv. Mater..

[59] Han W., Qin X., Wu J., Li Q., Liu M., Xia Y., Du H., Li B., Kang F. (2018). Electrosprayed porous Fe_3_O_4_/carbon microspheres as anode materials for high-performance lithium-ion batteries. Nano Res..

[60] Park J.-S., Kang Y.C. (2017). Multicomponent (Mo, Ni) metal sulfide and selenide microspheres with empty nanovoids as anode materials for Na-ion batteries. J. Mater. Chem. A.

[61] Deng L., Yang Z., Tan L., Zeng L., Zhu Y., Guo L. (2018). Investigation of the Prussian Blue Analog Co_3_[Co(CN)_6_]_2_ as an Anode Material for Nonaqueous Potassium-Ion Batteries. Adv. Mater..

[62] Park G.D., Kang Y.C. (2016). One-pot synthesis of CoSex–rGO composite powders by spray pyrolysis and their application as anode material for sodium-ion batteries. Chem. Eur. J..

[63] Qin W., Chen T., Lu T., Chua D., Pan L. (2016). Layered nickel sulfide-reduced graphene oxide composites synthesized via microwave-assisted method as high performance anode materials of sodium-ion batteries. J. Power Sources.

[64] Hu L., Huang Y., Zhang F., Chen Q. (2013). CuO/Cu_2_O composite hollow polyhedrons fabricated from metal–organic framework templates for lithium-ion battery anodes with a long cycling life. Nanoscale.

[65] Wang Z., Wang Z., Liu W., Xiao W., Lou X. (2013). Amorphous CoSnO_3_@C nanoboxes with superior lithium storage capability. Energy Environ. Sci..

[66] Wang H., Jiang Y., Manthiram A. (2019). N-doped Fe_3_C@C as an efficient polyselenide reservoir for high-performance sodium-selenium batteries. Energy Storage Mater..

[67] Oh S., Kim H., Kwon Y., Kim M., Cho E., Kwon H. (2016). Porous Co–P foam as an efficient bifunctional electrocatalyst for hydrogen and oxygen evolution reactions. J. Mater. Chem. A.

[68] Stern L.-A., Feng L., Song F., Hu X. (2015). Ni_2_P as a Janus catalyst for water splitting: The oxygen evolution activity of Ni_2_P nanoparticles. Energy Environ. Sci..

[69] Yan Y., Thia L., Xia B., Ge X., Liu Z., Fisher A., Wang X. (2015). Construction of Efficient 3D Gas Evolution Electrocatalyst for Hydrogen Evolution: Porous FeP Nanowire Arrays on Graphene Sheets. Adv. Sci..

[70] Fang D., Sun P., Huang S., Shang Y., Li X., Yan D., Lim Y., Su C.-Y., Su B.-J., Juang J.-Y. (2021). An Exfoliation–Evaporation Strategy To Regulate N Coordination Number of Co Single-Atom Catalysts for High-Performance Lithium–Sulfur Batteries. ACS Mater. Lett..

[71] Chen X., Zeng S., Muheiyati H., Zhai Y., Li C., Ding X., Wang L., Wang D., Xu L., He Y. (2019). Double-shelled Ni–Fe–P/N-doped carbon nanobox derived from a prussian blue analogue as an electrode material for K-ion batteries and Li–S batteries. ACS Energy Lett..

[72] Zhao L.F., Hu Z., Lai W., Tao Y., Peng J., Miao Z., Wang Y., Chou S., Liu H., Dou S. (2021). Hard carbon anodes: Fundamental understanding and commercial perspectives for Na-ion batteries beyond Li-ion and K-ion counterparts. Adv. Energy Mater..

[73] Shi L., Li D., Yao P., Yu J., Li C., Yang B., Zhu C., Xu J. (2018). SnS_2_ Nanosheets Coating on Nanohollow Cubic CoS_2_/C for Ultralong Life and High Rate Capability Half/Full Sodium-Ion Batteries. Small.

[74] Li W.-J., Chou S.-L., Wang J.-Z., Liu H.-K., Dou S.-X. (2015). A new, cheap, and productive FeP anode material for sodium-ion batteries. Chem. Commun..

[75] Shi S., Li Z., Shen L., Yin X., Liu Y., Chang G., Wang J., Xu S., Zhang J., Zhao Y. (2020). Electrospun free-standing FeP@NPC film for flexible sodium ion batteries with remarkable cycling stability. Energy Storage Mater..

[76] Shi S., Sun C., Yin X., Shen L., Shi Q., Zhao K., Zhao Y., Zhang J. (2020). FeP Quantum Dots Confined in Carbon-Nanotube-Grafted P-Doped Carbon Octahedra for High-Rate Sodium Storage and Full-Cell Applications. Adv. Funct. Mater..

[77] Wang C., Yan J., Li T., Lv Z., Hou X., Tang Y., Zhang H., Zheng Q., Li X. (2021). A Coral-Like FeP@ NC Anode with Increasing Cycle Capacity for Sodium-Ion and Lithium-Ion Batteries Induced by Particle Refinement. Angew. Chem. Int. Ed..

[78] Lim C.S., Sofer Z., Mazánek V., Pumera M. (2015). Layered titanium diboride: Towards exfoliation and electrochemical applications. Nanoscale.

[79] Carenco S., Portehault D., Boissiere C., Mezailles N., Sanchez C. (2013). Nanoscaled metal borides and phosphides: Recent developments and perspectives. Chem. Rev..

[80] Dutta S., Han H., Je M., Choi H., Kwon J., Park K., Indra A., Kim K., Paik U., Song T. (2020). Chemical and structural engineering of transition metal boride towards excellent and sustainable hydrogen evolution reaction. Nano Energy.

[81] Xu N., Cao G., Chen Z., Kang Q., Dai H., Wang P. (2017). Cobalt nickel boride as an active electrocatalyst for water splitting. J. Mater. Chem. A.

[82] Masa J., Sinev I., Mistry H., Ventosa E., De La Mata M., Arbiol J., Muhler M., Cuenya B.R., Schuhmann W. (2017). Ultrathin high surface area nickel boride (NixB) nanosheets as highly efficient electrocatalyst for oxygen evolution. Adv. Energy Mater..

[83] Elumeeva K., Masa J., Medina D., Ventosa E., Seisel S., Kayran Y., Genç A., Bobrowski T., Weide P., Arbiol J. (2017). Cobalt boride modified with N-doped carbon nanotubes as a high-performance bifunctional oxygen electrocatalyst. J. Mater. Chem. A.

[84] Chen P., Xu K., Zhou T., Tong Y., Wu J., Cheng H., Lu X., Ding H., Wu C., Xie Y. (2016). Strong-coupled cobalt borate nanosheets/graphene hybrid as electrocatalyst for water oxidation under both alkaline and neutral conditions. Angew. Chem. Int. Ed..

[85] Xu X., Liang H., Ming F., Qi Z., Xie Y., Wang Z. (2017). Prussian blue analogues derived penroseite (Ni, Co) Se_2_ nanocages anchored on 3D graphene aerogel for efficient water splitting. ACS Catal..

[86] Jiang Y., Fang Y., Chen C., Ni P., Kong B., Song Z., Lu Y., Niu L. (2019). Amorphous cobalt boride nanosheets directly grown on nickel foam: Controllable alternately dipping deposition for efficient oxygen evolution. ChemElectroChem.

[87] Guo F., Wu Y., Chen H., Liu Y., Yang L., Ai X., Zou X. (2019). High-performance oxygen evolution electrocatalysis by boronized metal sheets with self-functionalized surfaces. Energy Environ. Sci..

[88] An L., Sun Y., Zong Y., Liu Q., Guo J., Zhang X. (2018). Nickel iron boride nanosheets on rGO for active electrochemical water oxidation. J. Solid State Chem..

[89] Ma X., Meng H., Cai M., Shen P. (2012). Bimetallic carbide nanocomposite enhanced Pt catalyst with high activity and stability for the oxygen reduction reaction. J. Am. Chem. Soc..

[90] Naguib M., Halim J., Lu J., Cook K., Hultman L., Gogotsi Y., Barsoum M. (2013). New two-dimensional niobium and vanadium carbides as promising materials for Li-ion batteries. J. Am. Chem. Soc..

[91] Xiao Y., Hwang J.-Y., Sun Y.-K. (2016). Transition metal carbide-based materials: Synthesis and applications in electrochemical energy storage. J. Mater. Chem. A.

[92] Lukatskaya M.R., Mashtalir O., Ren C., Dall’Agnese Y., Rozier P., Taberna P., Naguib M., Simon P., Barsoum M., Gogotsi Y. (2013). Cation intercalation and high volumetric capacitance of two-dimensional titanium carbide. Science.

[93] Zou H., He B., Kuang P., Yu J., Fan K. (2018). Metal–Organic Framework-Derived Nickel–Cobalt Sulfide on Ultrathin Mxene Nanosheets for Electrocatalytic Oxygen Evolution. ACS Appl. Mater. Interfaces.

[94] Li J., Liu H., Sun K., Wang R., Qian C., Yu F., Zhang L., Bao W. (2022). Dual-functional iodine photoelectrode enabling high performance photo-assisted rechargeable lithium iodine batteries. J. Mater. Chem. A.

[95] Itaya K., Uchida I., Neff V. (1986). Electrochemistry of polynuclear transition metal cyanides: Prussian blue and its analogues. Acc. Chem. Res..

[96] Matsuda T., Moritomo Y. (2012). Two-electron reaction without structural phase transition in nanoporous cathode material. J. Nanotechnol..

[97] Bauer A., Song J., Vail S., Pan W., Barker J., Lu Y. (2018). The scale-up and commercialization of nonaqueous Na-ion battery technologies. Adv. Energy Mater..

[98] Moritomo Y., Takachi M., Kurihara Y., Matsuda T. (2012). Thin film electrodes of Prussian blue analogues with rapid Li^+^ intercalation. Appl. Phys. Express.

[99] Ma F., Li Q., Wang T., Zhang H., Wu G. (2017). Energy storage materials derived from Prussian blue analogues. Sci. Bull..

[100] Bao W., Wang R., Li B., Qian C., Zhang Z., Li J., Liu F. (2021). Stable alkali metal anodes enabled by crystallographic optimization—A review. J. Mater. Chem. A.

